# Atorvastatin suppresses NLRP3 inflammasome activation in intracerebral hemorrhage via TLR4- and MyD88-dependent pathways

**DOI:** 10.18632/aging.203824

**Published:** 2022-01-11

**Authors:** Duo Chen, Lu Sui, Cao Chen, Sanchuan Liu, Xianfeng Sun, Junhong Guan

**Affiliations:** 1 Department of Neurosurgery, Shengjing Hospital of China Medical University, Shenyang 110004, PR China; 2 Department of Pathophysiology, Shenyang Medical College, Shenyang 110034, PR China

**Keywords:** intracerebral hemorrhage, atorvastatin, toll-like receptor 4, NLRP3 inflammasome, neuronal loss

## Abstract

Intracerebral hemorrhage (ICH) is a common neurological condition that causes severe disability and even death. Even though the mechanism is not clear, increasing evidence shows the efficacy of atorvastatin on treating ICH. In this study, we examined the impact of atorvastatin on the NOD-like receptor protein 3 (NLRP3) inflammasome and inflammatory pathways following ICH. Mouse models of ICH were established by collagenase injection in adult C57BL/6 mice. IHC mice received atorvastatin treatment 2 h after hematoma establishment. First, the changes of glial cells and neurons in the brains of ICH patients and mice were detected by immunohistochemistry and western blotting. Second, the molecular mechanisms underlying the microglial activation and neuronal loss were evaluated after the application of atorvastatin. Finally, the behavioral deficits of ICH mice without or with the treatment of atorvastatin were determined by neurological defect scores. The results demonstrated that atorvastatin significantly deactivated glial cells by reducing the expression of glial fibrillary acidic protein (GFAP), Ionized calcium binding adapter molecule 1 (Iba1), tumor necrosis factor (TNF)-α, and interleukin (IL)-6 in ICH model mice. For inflammasomes, atorvastatin also showed its efficacy by decreasing the expression of NLRP3, cleaved caspase-1, and IL-1β in ICH mice. Moreover, atorvastatin markedly inhibited the upregulation of toll-like receptor 4 (TLR4) and myeloid differentiation factor 88 (MyD88), which indicated deactivation of NLRP3 inflammasomes. By inhibiting the activities of inflammasomes in glial cells, neuronal loss was partially prevented by suppressing the apoptosis in the brains of ICH mice, protecting them from neurological defects.

## INTRODUCTION

Intracerebral hemorrhage (ICH), a subtype of stroke, is characterized by high incidence (24.6 per 100000 person-years), fatality (40.4% at 1 month), and disability rates and affects millions of people worldwide [1]. Fifty percent of ICH survivors develop disabilities, placing a heavy burden on their families and society [2]. Neurological impairment and cerebral injury are observed 2–3 d after the event [3, 4]. After ICH, microglia and astrocytes co-function to induce the secondary inflammatory response [5]. Microglial cells release proinflammatory cytokines, such as interleukin (IL)-6, tumor necrosis factor (TNF)-α [6], and IL-1β [7], causing neuronal death and exacerbating nerve damage [8].

Four subtypes of related proteins, NOD-like receptor protein (NLRP)1, NLRP3, NLRP4 and AIM2, have been identified that facilitate the formation of vacuoles [9], of which the NLRP3 inflammasome is the most widely characterized [10]. NLRP3 can activate caspase-8, which subsequently cleaves pro-IL-1β into its mature form [11], thereby making the NLRP3 inflammasome the major IL-1β-converting protease [6, 12, 13]. In this way, the NLRP3 inflammasome contributes to inflammation after ICH [14]. Conversely, specifically inhibiting the activity of NLRP3 inflammasome reduces brain injury after ICH [15]. Knocking down the expression of NLRP3 by recombinant adenovirus attenuates inflammation and brain injury after ICH [16]. From these observations, dysregulated NLRP3 inflammasome activation may be involved in regulating the development and progression of ICH.

Given the potential roles of NLRP3 inflammasome in ICH, accumulating evidence indicates that toll like receptor 4 (TLR4) mediates the effects of the NLRP3 inflammasome on activating macrophages and monocytes [17, 18]. Except for NLRP3 inflammasomes, TLR4 plays an important role in immunological recognition and inflammation regulation in myeloid differentiation factor 88 (MyD88)-dependent and –independent mechanisms [19, 20]. Moreover, the changes in microglial polarization are regulated by TLR4 and NF-κB pathway-associated proteins [21, 22]. Unlike other TLRs, TLR4 is implicated in ICH [23–25]. TLR4 is stimulated in ICH and the downstream NF-kB signal therefore activated, promoting the expression of inflammatory factors [26]. Considering the above evidence, TLR4 might be involved in the molecular mechanisms of NLRP3 inflammasomes on ICH.

Until now, little progress has been made in treating the subsequent inflammatory cascade, despite advances in surgical techniques addressing primary brain injury caused by ICH [27]. As a 3-hydroxy-3-methyglutaryl coenzyme A (HMG-CoA) inhibitor, atorvastatin effectively decreases the levels of low-density lipoprotein and cholesterol. Atorvastatin treatment also produces greater benefits than expected from solely reducing lipid levels [28, 29]. One of these beneficial effects is its anti-inflammatory properties [30, 31]. Therefore, the current study aimed to determine whether atorvastatin regulates the expression of the NLRP3 inflammasome during ICH, and the potential underlying molecular mechanisms.

## MATERIALS AND METHODS

### Chemicals and antibodies

Atorvastatin, lipopolysaccharide (LPS), Bay11-7082, JSH-23, and mIL-1β were obtained from Sigma-Aldrich (Shanghai, China). Antibodies specific for Iba1 (#17198), GFAP (#80788), NeuN (#24307), IL-1β (#12242), TNF-α (#3707), IL-6 (#12912 and 12153), NLRP3 (#15101), ASC (#13833 and 67824), cleaved caspase-1 (#89332 and 4199), p-NF-κB (#8242), TLR4 (#14358), MyD88 (#4283), Bax (#14796), Bcl-2 (#3498), and GAPDH (#5174) were purchased from Cell Signaling Technology (Shanghai, China). Antibody specific for TLR4 (#ab22048) was obtained from Abcam (Shanghai, China).

### Brain samples

Brain tissue of deceased ICH patients was collected from the Department of Neurosurgery, Shengjing Hospital of China Medical University (Shenyang, Liaoning, China). The corresponding control tissue was collected from the non-affected part of the same ICH patients. All experimental protocols were approved by the ethics committee of the China Medical University (2021PS116K). Half of the sample was fixed in paraformaldehyde, and the other half was stored at −80°C.

### Animals and groups

Male C57BL/6 mice (20–30 g each) were obtained from Liaoning Changsheng Biotechnology Co., Ltd. (Benxi, Liaoning, China). The mice were kept in a temperature-controlled room with a 12 h light/12 h dark cycle and provided with food and water. The mice were divided into 5 groups of 8 each: sham, ICH group, and three drug treatment groups (10, 20, or 40 mg/kg atorvastatin). All experimental procedures were conducted in accordance with the Guidance of Care and Use of Laboratory Animals and approved by the ethics committee of the China Medical University (2021PS257K).

### Animal model

Collagenase injection was applied to induce ICH in the experimental mice. Briefly, the mice were anesthetized with isoflurane (Solarbio Life Sciences, Beijing, China) using a suction mask (Kopf Instruments, Tujunga, California). The skull was incised at the center of the scalp after shaving the fur. Collagenase VII-S (0.075 U in 0.5 μL saline; Sigma-Aldrich, Shanghai, China) was injected into the right striatum using a 26-pin stereotactic needle adjusted to anterior 0.8 mm, lateral 2.0 mm, and ventral 3.6 mm of the bregma. After injection, the needle was slowly removed to avoid the loss of solution. The sham group underwent all the surgical procedures without collagenase injection. After closing the incision, the mice were returned to their cages for recovery.

### Drug administration

Two hours after inducing ICH, mice were intragastrically administered 10, 20, or 40 mg/kg of atorvastatin once daily for 7 days. The sham groups were administered with the same volume of vehicle solvent. Neurological defects were evaluated, and then the mice were sacrificed with CO_2_ to harvest brain tissue.

### Evaluation of neurological defect scores

The neurological defect scores were determined by the Longa scoring method at days 1, 3, and 7 of atorvastatin administration as previously described [32]. The scoring definitions were as follows: 0–mice moved freely and symmetrically; 1–mice lifted their tails but could not completely stretch left front paws; 2–mice could not stretch the left front paws completely and their left forelimb did not function smoothly; 3–the left forelimb clung to the chest; 4–movements were unconsciously left-oriented; 5–the left forepaw was pulled back when the mouse turned left; 6–mice circled to the left; 7–mice could not support themselves and fell down to the left.

### Assessment of cerebral edema

Brain water content (BWC) was measured as described in previous studies [33] to quantify brain edema from the weights of both dry and wet brains. In brief, mice were sacrificed, after which wet weight (WW) of the brain was measured. The dry weight (DW) was obtained by drying the samples at 100°C for 24 h in an electric oven. The BWC was calculated by the following equation:


$$BWC=\frac{WW-DW}{WW}\times 100\%$$

### Immunohistochemistry (IHC)

Brain tissues were immediately submerged in 4% paraformaldehyde, embedded with paraffin, and sectioned with a microtome. After rehydration, endogenous peroxidase was blocked by incubation in 3% H_2_O_2_ for 10 min. Antigen cross-linking was performed in an autoclave for 3 min. All preparations were treated with goat serum to block non-specific binding for 45 min. Primary antibodies were applied at a dilution of 1:100 for one hour, and the sections were stained using an avidin biotin-peroxidase technique. The reaction was developed with diaminobenzidine solution (Abcam, Shanghai, China) for 10 min. Negative control sections were stained using the same steps, but the primary antibody was omitted.

### Nissl staining

Nissl staining was performed to determine the number of surviving neurons. The sections were incubated with Nissl staining solution at 40°C for 10 min, then washed with 95% ethanol and 70% alcohol. Images were obtained with a light microscope (BX51; Olympus, Tokyo, Japan).

### Cell culture and treatments

Mouse BV2 and neuroblastoma (N) 2a cells were purchased from the American Type Culture Collection (ATCC) and cultured in Dulbecco’s Modified Eagle Medium (DMEM, Life Technologies, Shanghai, China) supplemented with 10% fetal bovine serum (Life Technologies). For experiments, BV2 cells were treated with LPS (100 ng/mL) in the absence or presence of atorvastatin (10, 20, or 40 μM), Bay11-7082 (2 μM) or JSH-23 (10 μM). N2a cells were treated with IL-1β (10 ng/mL) in the presence or absence of Bay11-7082 (2 μM) or JSH-23 (10 μM). After treatment, cells were lysed for western blotting.

### siRNA transfection

BV2 cells were transfected with 100 nM of siRNA for 8 h with siRNA transfection reagent (RiboBio, Guangzhou, Guangdong, China) to knockdown the expression of TLR4. Briefly, cells were treated with siRNA duplex solution for 12 h after washing with fresh medium without antibiotics. The medium was subsequently replaced with culture medium. Control cells were transfected with scrambled siRNA sequence (RiboBio, Guangzhou, China). Cell lysates were utilized for western blotting to verify the efficacy of protein knockdown.

### Western blotting

Mouse brain tissue, human brain tissue, and cultured cells (collected after washing with PBS) were each placed in ice-cold radioimmunoprecipitation assay (RIPA) lysis buffer containing phenylmethylsulfonyl fluoride (PMSF) (RIPA: PMSF = 100:1) for 30 min, homogenized using a tissue grinder, and centrifuged at 10,000 rpm for 20 min at 4°C. The supernatants were collected and total protein concentrations were measured by the BCA method (Beyotime, Shanghai, China). After dilution with loading buffer, proteins were subjected to SDS-PAGE and transferred to polyvinylidene fluoride, which was then blocked with 5% skim milk for 1 h, and probed with different primary antibodies (1:1000, v/v) at room temperature for 1 h. The membranes were then incubated with secondary antibody (1:10,000, v/v) for 1 h at room temperature. The bands were visualized by enhanced chemiluminescence and observed under ChemiDoc imaging instrument (Bio-Rad Laboratories, Shanghai, China). The optical density of the bands was analyzed using Image J software. GAPDH served as an internal control.

### Enzyme-linked immunosorbent assay (ELISA)

Mouse or human brain tissue (10 mg) was pulverized in liquid nitrogen and centrifuged at 4°C to collect supernatants. Standard, black, and sample testing wells were designated and after diluting the samples with chemiluminescence reagents, 100 μL was added to the corresponding wells. The concentrations of IL-1β, TNF-α, and IL-6 were determined by the absorbance at a wavelength of 450 nm using ELISA kits according to the manufacturer’s instructions (Beyotime, Shanghai China/Solarbio Life Sciences, Beijing, China).

### Statistics

All results are based on at least three separate experiments and are expressed as mean ± standard deviation (SD). Two-way analysis of variance (ANOVA) with least significant difference (LSD) post hoc analysis was used to analyze BWC and neurobehavior. Other data were statistically compared using one-way ANOVA with LSD post hoc analysis. We considered *P* < 0.05 statistically significant.

## RESULTS

### Neuroinflammation and neuronal loss were induced in ICH patients

**Figure 1:**
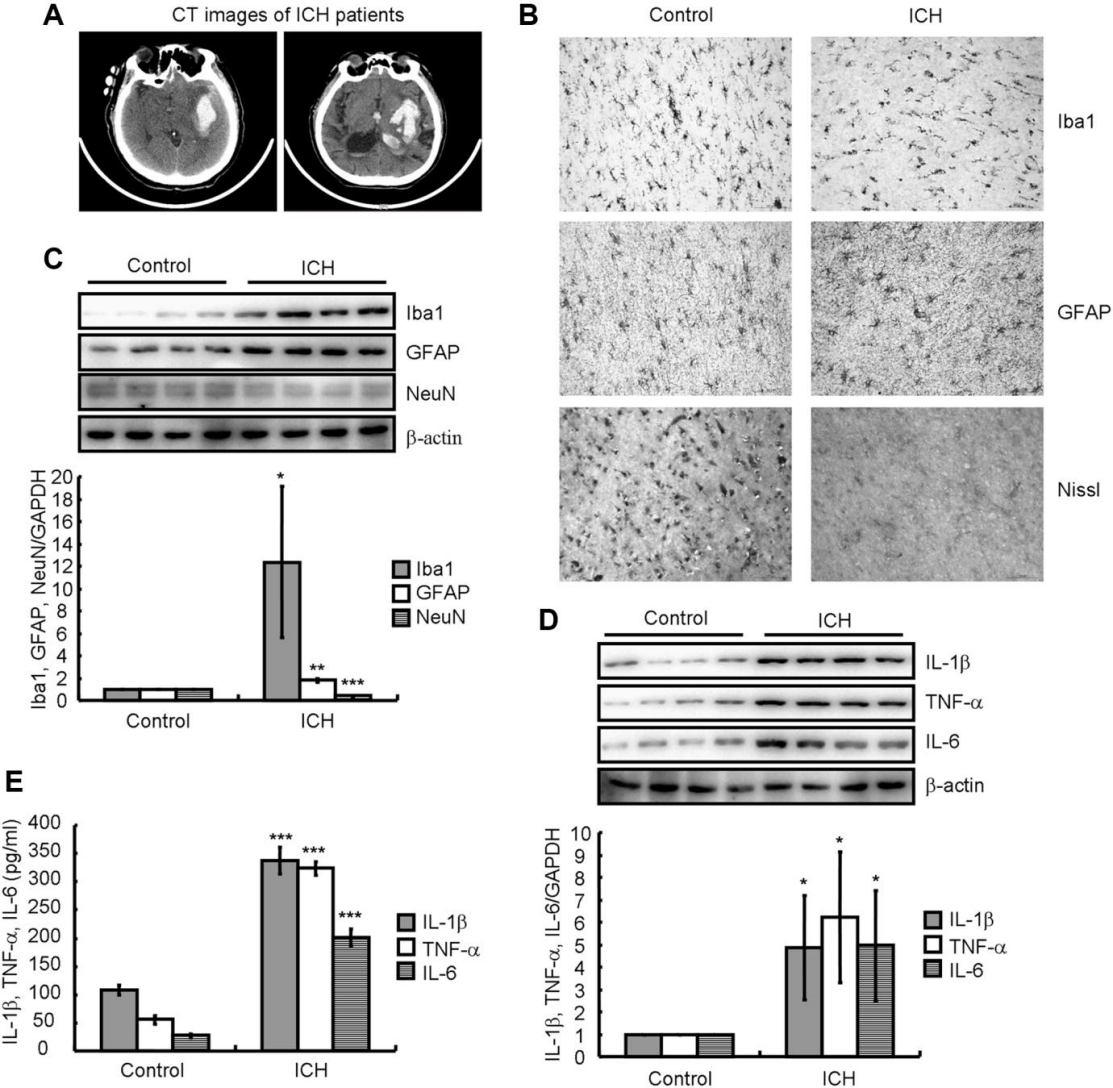
**Neuroinflammation and neuronal loss were present in ICH patients.** (**A**) A computed tomography (CT) image indicates hematoma formation in an ICH patient compared to a control subject. (**B**) Paraffin slices immunostained for Iba1 or GFAP show microglia and astrocytes, respectively. The morphology of neurons is revealed by Nissl staining. (**C**, **D**) Western blotting detects the protein expression of Iba1, GFAP, NeuN, IL-1β, TNF-α, and IL-6 in a patient with ICH. GAPDH serves as an internal control. (**E**) Concentrations of IL-1β, TNF-α, and IL-6 in the brain tissues of ICH patients were detected by ELISA. The results represent the mean ± SD for the repeated experiments. ^*^P < 0.05; ^**^P < 0.01; ^***^P < 0.001 vs. control subjects.

The brain tissues of ICH patients and corresponding control subjects from the non-affected part of the same ICH patients were examined for neuroinflammation and neuronal loss. As shown in Figure 1A, computed tomography (CT) showed hematoma formation in ICH patients. To explore the damaging effects on adjacent tissues, microglia and astrocytes were labeled with Iba1 and GFAP, respectively, and neuronal loss was analyzed using IHC and western blotting. We found that when compared with controls, Nissl staining revealed neuronal loss (Figure 1B), and there was a significant increase in Iba1 and GFAP protein levels and decrease in NeuN protein levels in ICH patients (Figure 1C). Additionally, the levels of IL-1β, TNF-α, and IL-6 in ICH patients were markedly higher than that in control subjects (Figure 1D, 1E). Therefore, we hypothesized that the induction of neuroinflammation resulted in neuronal loss in ICH patients.

### NLRP3 inflammasomes were activated in ICH patients

**Figure 2:**
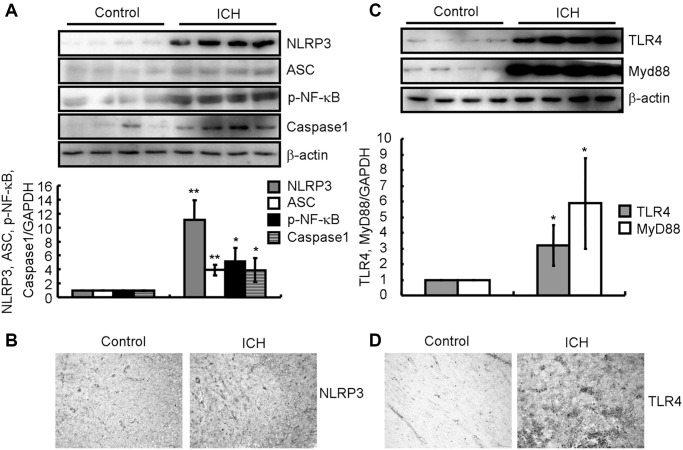
**NLRP3 inflammasomes and TLR4 signaling pathways were activated in ICH patients.** (**A**, **B**) Western blotting and IHC were used to analyze the protein expression of NLRP3, ASC and cleaved caspase-1 as well as the phosphorylation of NF-κB in the brains of ICH patients. GAPDH serves as an internal control. The band densities were measured by Image J to estimate protein levels. (**C**, **D**) Western blotting and IHC were used to analyze the protein expression of TLR4 and MyD88 in the brains of ICH patients. GAPDH serves as an internal control. The band densities were measured by Image J to estimate protein levels. The results represent the mean ± SD for the repeated experiments. ^*^P < 0.05; ^**^P < 0.01 vs. control subjects.

As IL-1β activation and secretion are primarily mediated by the cysteine protease caspase-1, which is activated by the inflammasome [12], we determined the expression of NLRP3, ASC and caspase-1 as well as the phosphorylation of NF-κB in the brains of ICH patients. By western blotting analysis, we found that the protein levels of NLRP3 and cleaved caspase-1 were significantly higher in ICH patients than in control subjects (Figure 2A). We then examined the changes in NLRP3 expression in the brain tissues around the ICH site. IHC revealed that the expression of NLRP3 inflammasome was clearly increased in the brains of ICH patients compared to that in control subjects (Figure 2B). Therefore, NLRP3 inflammasome activation might underly the induction of neuroinflammation in ICH patients.

### TLR4 and MyD88 signaling pathways were affected in ICH patients

As TLR4 has been reported to be implicated in ICH [23–25], we measured the expression of TLR4 and MyD88 in the brains of ICH patients. By western blotting analysis, we found that TLR4 and MyD88 protein levels were higher in ICH patients than in control subjects (Figure 2C). We then examined the changes in TLR4 expression in the brain tissues around the ICH site. IHC revealed that TLR4 expression was noticeably higher in ICH patients (Figure 2D). Therefore, TLR4 activation might be involved in regulating neuroinflammation in ICH patients.

### ICH induces neuroinflammation and neuronal loss via activation of TLR4-dependent NLRP3 inflammasome-activating mechanisms in collagenase injected mice

**Figure 3:**
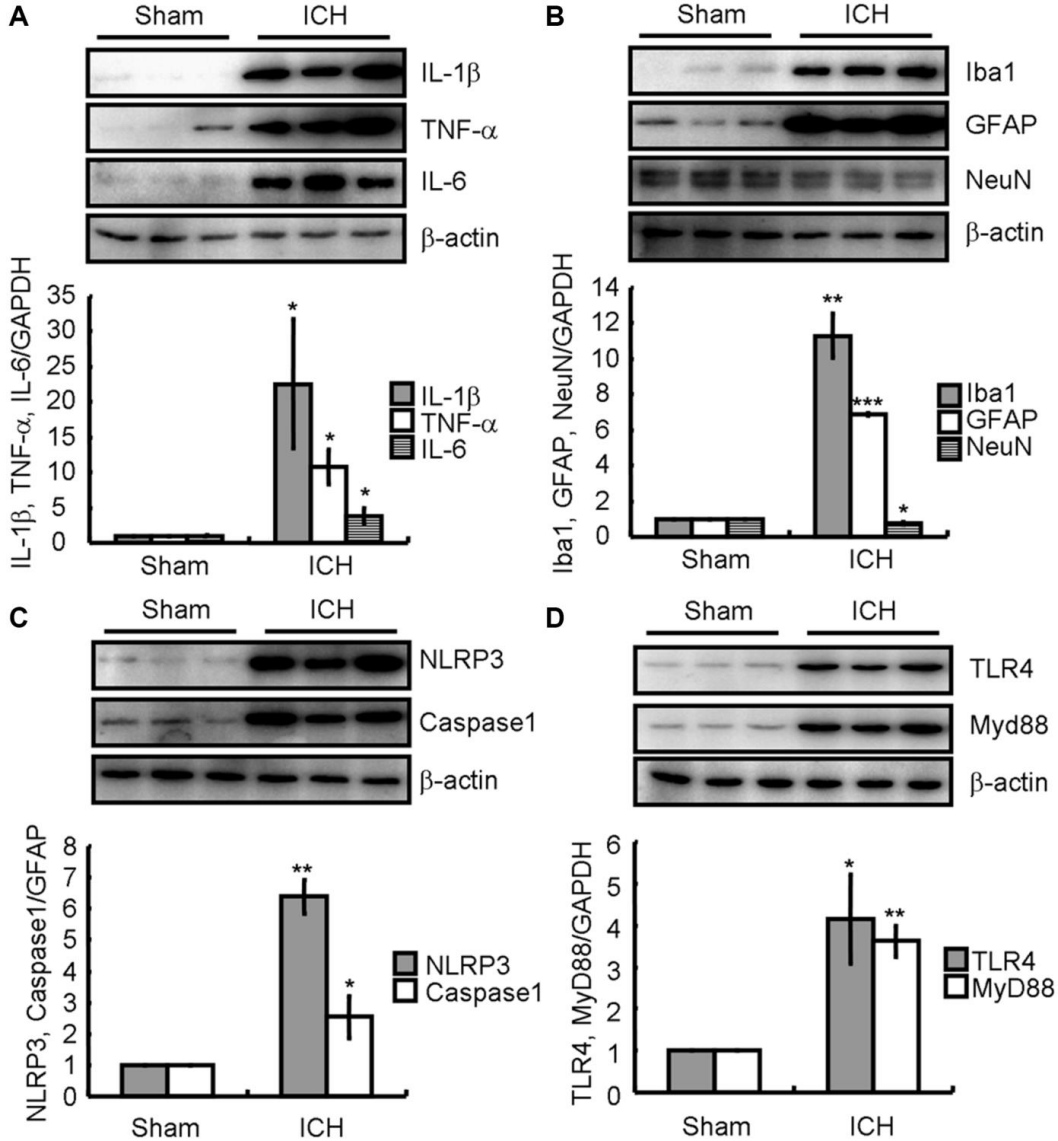
**ICH induces neuroinflammation and neuronal loss by activating TLR4-dependent NLRP3 inflammasomes-activating mechanisms in the collagenase injected mice.** (**A**) IL-1β, TNF-α, and IL-6; (**B**) Iba1, GFAP, and NeuN; (**C**) NLRP3 and caspase-1; (**D**) TLR4 and MyD88 protein expression were determined by western blotting analysis. GAPDH served as an internal control. The band densities were measured by Image J to estimate protein levels. The results represent the mean ± SD for the repeated experiments. ^*^P < 0.05; ^**^P < 0.01; ^***^P < 0.001 vs. sham animals.

To further reveal the inherent mechanisms of ICH, animal models were established by injecting collagenase VII-S to the right striatum to form a hematoma, and the brains of ICH and sham groups were collected after 7 days to examine neuroinflammation and neuronal loss. Western blotting revealed that the levels of IL-1β, TNF-α, and IL-6 in the brains of ICH-induced mice were markedly higher than in the sham group (Figure 3A). Moreover, there was a significant increase in Iba1 and GFAP and decrease in NeuN protein levels (Figure 3B). Additionally, we determined that the NLRP3 and cleaved caspase-1 protein levels were significantly higher in ICH than sham animals (Figure 3C). Finally, the TLR4 and MyD88 protein levels were clearly elevated in the ICH group (Figure 3D). Thus, the properties of this ICH animal model were similar to that of patients with ICH and suitable for subsequent experiments.

### Atorvastatin attenuates the effects of ICH on neuroinflammation induction and neuronal loss

**Figure 4:**
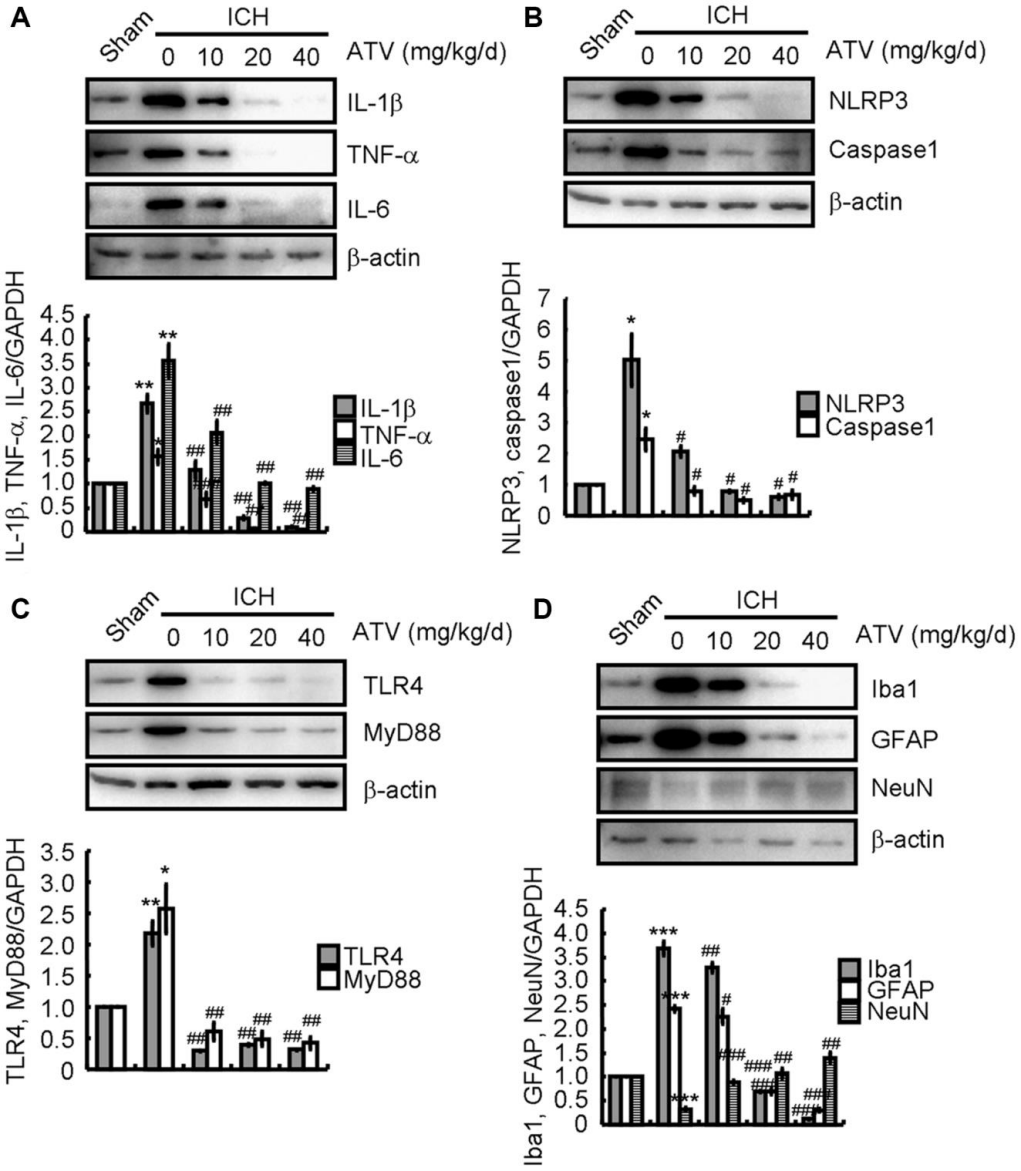
**Atorvastatin attenuates the effects of ICH on inducing neuroinflammation and neuronal loss.** The ICH mice were treated with the indicated concentration of atorvastatin for 7 d. Total proteins were extracted by RIPA buffer. (**A**) IL-1β, TNF-α and IL-6; (**B**) NLRP3 and caspase1; (**C**) TLR4 and MyD88; and (**D**) Iba1, GFAP and NeuN protein expression were determined by western blotting analysis. GAPDH served as an internal control. The band densities were measured by Image J to estimate protein quantities. The results represent the mean ± SD for the repeated experiments. ^*^P < 0.05; ^**^P < 0.01; ^***^P < 0.001 vs. sham animals. ^#^*P* < 0.05; ^##^*P* < 0.01; ^###^*P* < 0.001 vs. ICH only animals.

Since atorvastatin has beneficial anti-inflammatory properties [30, 31], ICH mice were treated with the indicated concentrations of atorvastatin for 7 d after ICH induction. As shown in Figure 4A, treatment with different atorvastatin concentrations (10–40 mg/kg/d) dose-dependently antagonized the ICH-induced increase in IL-1β, TNF-α, and IL-6 protein levels. Atorvastatin treatment also significantly attenuated the ICH-induced increase in NLRP3 and cleaved caspase-1 levels (Figure 4B). Similarly, the TLR4 and MyD88 protein levels in atorvastatin-treated mice were lower than that in the ICH-only group (Figure 4C), suggesting that atorvastatin deactivates the TLR4 and MyD88 signaling pathways. Next, we profiled the variations of glial activation and neuronal loss around the ICH tissue without or with atorvastatin treatment. Western blotting revealed that the Iba1 and GFAP protein levels were decreased, whereas NeuN protein levels were restored in the atorvastatin-treated groups (Figure 4D), suggesting that atorvastatin could suppress ICH-induced neuroinflammation and neuronal loss.

**Figure 5:**
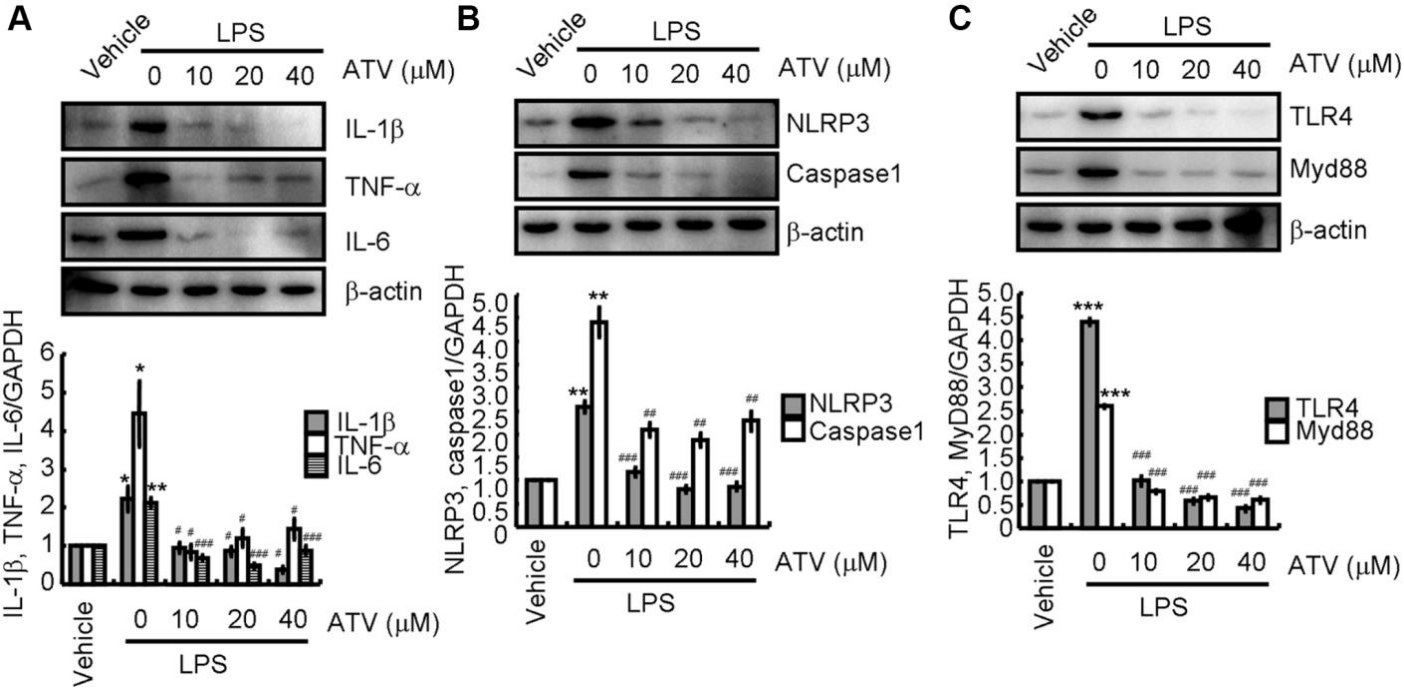
**Atorvastatin attenuates the effects of LPS on inducing neuroinflammation.** Total proteins were extracted from LPS-induced BV2 cells treated with atorvastatin. (**A**) IL-1β, TNF-α and IL-6; (**B**) NLRP3 and caspase-1; (**C**) TLR4 and MyD88 protein expression levels are revealed by western blotting analysis. GAPDH served as an internal control. The band densities were measured by Image J to estimate protein quantities. The results represent the mean ± SD for the repeated experiments. ^*^P < 0.05; ^**^P < 0.01; ^***^P < 0.001 vs. vehicle treatment. ^#^*P* < 0.05; ^##^*P* < 0.01; ^###^*P* < 0.001 vs. LPS treatment.

To validate the above observations *in vivo*, BV2 cells were treated with LPS in the absence or presence of atorvastatin. LPS stimulation significantly induced IL-1β, TNF-α, IL-6, NLRP3, cleaved caspase-1, TLR4, and MyD88 expression (Figure 5A–5C). After atorvastatin administration, IL-1β, TNF-α, IL-6, NLRP3, cleaved caspase-1, TLR4, and MyD88 protein levels were not markedly different from that in the non-treated controls, but were significantly lower than that in LPS-treated BV2 cells (Figure 5A–5C).

### TLR4 pathway activation induces NLRP3 inflammasome activation in ICH-induced neuroinflammation

**Figure 6:**
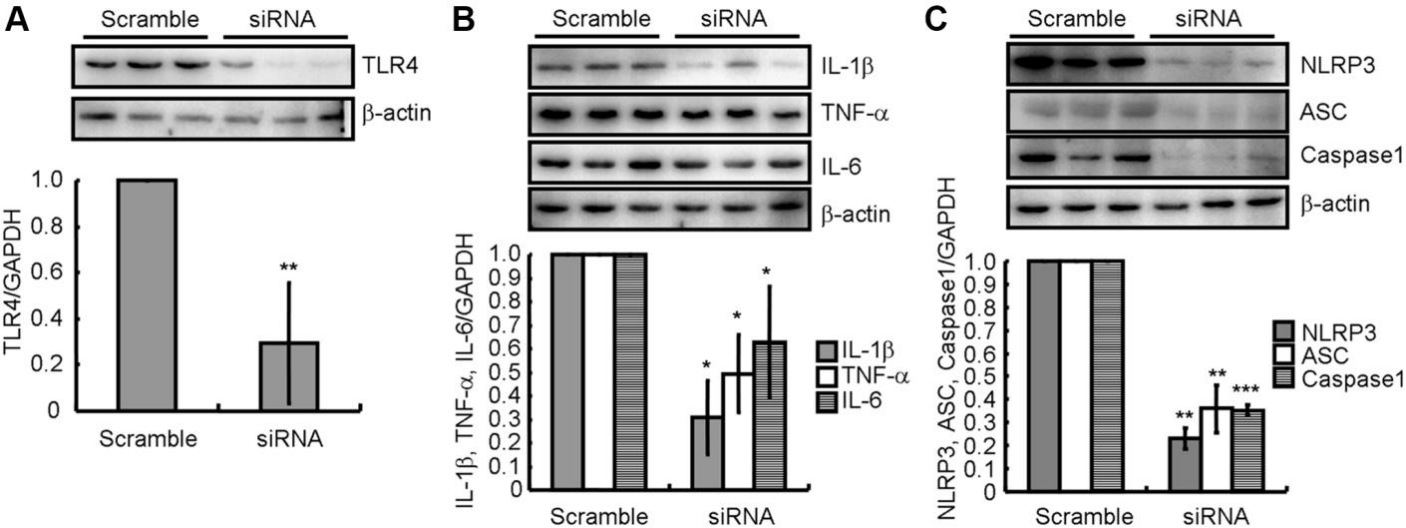
**TLR4 pathway activation participates in the activation of NLRP3 inflammasome in ICH-induced neuroinflammation.** (**A**) In TLR4 knocked-down BV2 cells, (**B**) IL-1β, TNF-α and IL-6; and (**C**) NLRP3, ASC and caspase-1 protein expression were determined by western blotting analysis. GAPDH served as an internal control. The band densities were measured by Image J and protein quantities estimated. The results represent the mean ± SD for the repeated experiments. ^*^P < 0.05; ^**^P < 0.01; ^***^P < 0.001 vs. scrambled siRNA.

To explore the mechanism associated with the effect of atorvastatin on the inflammasome, TLR4 siRNA was employed to disrupt the expression of TLR4 in LPS-treated BV2 cells. As shown in Figure 6A, siRNA efficiently knocked down TLR4 in BV2 cells. Moreover, western blotting revealed that IL-1β, TNF-α, and IL-6 expression was downregulated (Figure 6B). Presumably, the activity of NLRP3 inflammasomes were attenuated because of the decreased NLRP3, ASC and cleaved caspase-1 protein levels (Figure 6C). Thus, the TLR4 signaling pathway is critical for NLRP3 activation and pro-inflammatory cytokine expression. Furthermore, these observations also underscore the roles of NLRP3 inflammasomes in the production of pro-inflammatory cytokines.

### Activation of NLRP3 inflammasomes induces proinflammatory cytokine expression leading to neuronal apoptosis

**Figure 7:**
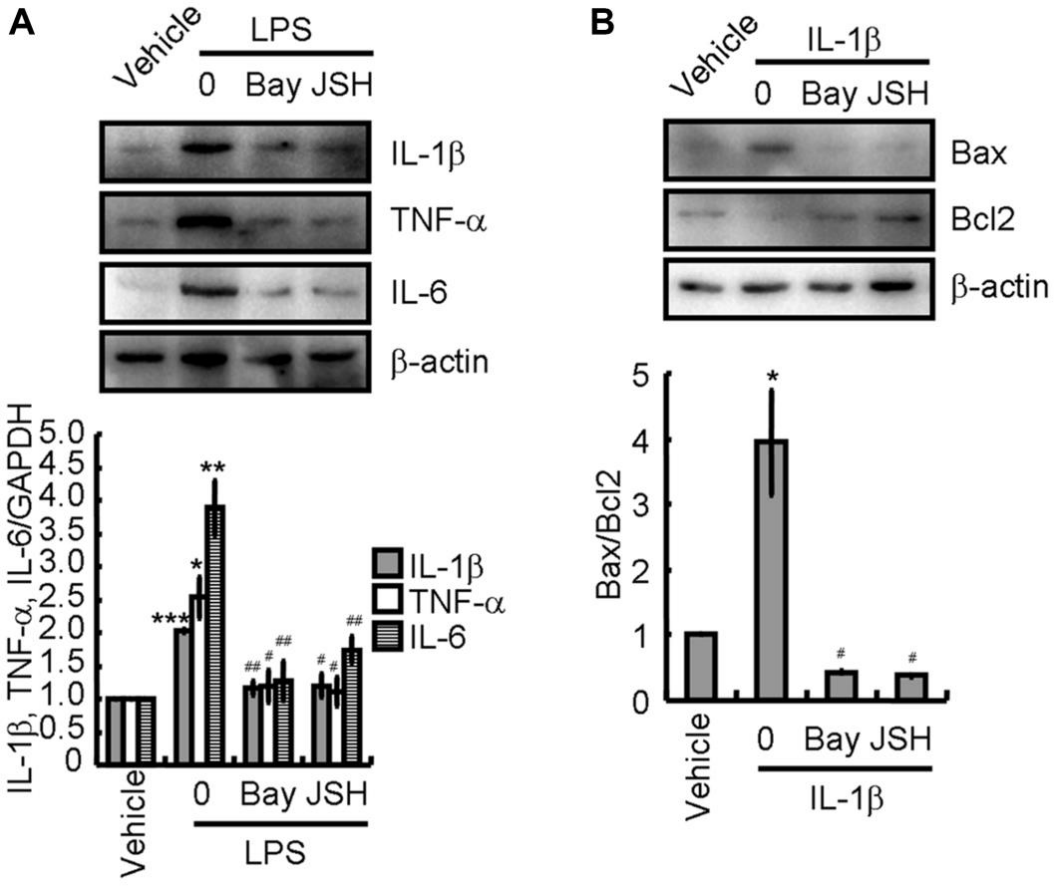
**Activation of NLRP3 inflammasomes is critical for inducing apoptosis of neurons.** (**A**) BV2 cells were treated with LPS without or with the indicated concentration of Bay11-7082 or JSH-23 for 24 h. IL-1β, TNF-α, and IL-6 protein expression were determined by western blotting. (**B**) N2a cells were treated with the IL-1β without or with the indicated concentration of Bay11-7082 or JSH-23 for 24 h. Bax and Bcl-2 protein expression were determined by western blotting analysis. GAPDH served as an internal control. The band densities were measured by Image J and protein quantities were estimated. The results represent the mean ± SD for the repeated experiments. ^*^P < 0.05; ^**^P < 0.01; ^***^P < 0.001 vs. vehicle treatment. ^#^*P* < 0.05; ^##^*P* < 0.01 vs. LPS or IL-1β treatment.

To study the mechanism underlying the reduction in pro-inflammatory cytokine production owing to atorvastatin, BV2 cells were pre-treated with NF-κB inhibitors, Bay11-7082 [34] or JSH-23 [35], for 30 min before addition of LPS. Complete inhibition of IL-1β, TNF-α, and IL-6 expression was observed, which was congruent with the levels in atorvastatin-treated BV2 cells (Figure 7A). This observation indicates that the inhibition of NLRP3 inflammasome activation mitigated the production of pro-inflammatory cytokines in LPS-activated BV2 cells. Furthermore, IL-1β was used to treat N2a cells with and without incubation with Bay11-7082 or JSH-23. Western blotting revealed that Bay11-7082 or JSH-23 remarkably abolished the effects of IL-1β on upregulation of Bax expression and downregulation of Bcl-2 expression, implying the occurrence of neuronal apoptosis (Figure 7B).

### Comparison of brain water content of mice

After a 7-day atorvastatin treatment, the brain tissues of the mice were removed and weighed to calculate their BWC. The BWC of the ICH group had increased significantly when compared with that of the sham group (Table 1). Compared with the ICH group, the BWC of the atorvastatin-treated groups were markedly decreased, while it showed no significant changes when compared to that in the sham group (Table 1), suggesting that atorvastatin treatment alleviated brain edema.

**Table 1:** Comparison of brain water content of mice in each group.

**Group**	**Sham**	**ICH**	**ICH+ATV (10 mg/kg)**	**ICH+ATV (20 mg/kg)**	**ICH+ATV (40 mg/kg)**
BWC	71.6 ± 0.9	79.1 ± 0.9^*^	74.0 ± 0.5^#^	73.3 ± 0.7^#^	73.0 ± 0.6^#^

### Comparison of neurological defect scores of each group

The neurological defect scores of mice in the sham group at days 1, 3, and 7 were consistently 0. Comparison of the neurological defect scores of the four experimental groups at day 1 did not show statistically significant differences. At days 3 and 7, the neurological defect scores of the atorvastatin-treated groups had decreased significantly when compared with those of the ICH only group (Table 2).

**Table 2:** Comparison of neurological defects scores of each group.

**Group**	**1st day**	**3rd day**	**7th day**
Sham	0.0 ± 0.0	0.0 ± 0.0	0.0 ± 0.0
ICH	4.6 ± 0.2^*^	3.8 ± 0.1^*^	3.3 ± 0.2^*^
ICH+ATV (10 mg/kg)	4.7 ± 0.1	2.5 ± 0.1^#^	1.6 ± 0.2^#^
ICH+ATV (20 mg/kg)	4.5 ± 0.1	2.6 ± 0.1^#^	1.6 ± 0.1^#^
ICH+ATV (40 mg/kg)	4.6 ± 0.1	2.4 ± 0.1^#^	1.0 ± 0.1^#^

## DISCUSSION

Accumulating evidence has revealed that neuroinflammation plays a critical role in ICH [6]. The expression of hallmark pro-inflammatory cytokines IL-1β, TNF-α, and IL-6 is likely associated with glial activation and neuronal loss [6]. Thus, targeting these proteins has been proven to effectively protect the brains from secondary inflammatory injury after ICH [27]. Moreover, NLRP3 inflammasomes manage the production of pro-inflammatory cytokines. The inactive precursor of IL-1β is proteolytically cleaved by caspase-1 upon its activation, which depends on NLRP3 inflammasome activation [36]. The NLRP3 inflammasome contributes to inflammation after ICH, as inhibition or knockdown of its activity reduces brain injury [14–16]. Here, we observed that atorvastatin inhibited NLRP3 inflammasome activation, the cleavage of caspase-1, and IL-1β expression in ICH via the TLR4- and MyD88-dependent pathways, providing evidence that atorvastatin inhibits neuronal loss. Therefore, we show a novel effect of atorvastatin on NLRP3 inflammasome activation suggesting that it may ameliorate the ICH-associated BWC and neurologic defects, providing a new therapeutic strategy.

Recent studies also suggest that statins have the ability to suppress neuroinflammation via deactivating inflammasomes. For instance, atorvastatin could inhibit NLRP3 inflammasomes activation and IL-1β secretion in PMA-stimulated THP-1 cells [18]. Moreover, rosuvastatin could suppress NLRP3 inflammasome and IL-1β release in rats with type 2 diabetes and alleviate diabetes-induced cardiac dysfunction [37]. However, conflicting effects of other statins on the activities of NLRP3 inflammasome are difficult to reconcile. Pravastatin pretreatment enhanced the NLRP3 inflammasome activation in LPS-primed macrophages and bone marrow-derived macrophages [38]. In addition, fluvastatin and lovastatin induced caspase-1 activation and IL-1β secretion in LPS-stimulated THP-1 cells [39]. In contrast, simvastatin does not show any effect on IL-1β secretion in PMA-activated human peripheral blood mononuclear cells (PBMCs) and induced caspase-1 activation and IL-1β secretion in LPS-activated PBMCs and THP-1 cells by inhibiting the mevalonate pathway [40]. These differences might be ascribed to different stimulus requirements for macrophages.

As the upstream initiator, TLR4 plays an important role in activating inflammation [41]. Within the TLR4 signaling pathway, the MyD88-dependent signaling pathway is an important activator of NF-κB and the subsequent regulatory effects of NF-κB signaling [42]. Of note, NF-κB is a key transcriptional factor for regulating the inflammatory response [43]. There are a series of studies indicating that NLRP3, IL-1β, and IL-6 are the target genes of NF-κB [41, 44, 45]. Consistently, our data also showed that Bay11-7082, an NF-κB specific inhibitor, reversed the LPS-induced expression of pro-inflammatory cytokines IL-1β, TNF-α, and IL-6, suggesting that NF-κB activation could participate in regulating neuroinflammation. In line with our results, the TLR4 signaling pathway is activated in ICH, leading to the activation of the downstream NF-κB pathway and the expression of different innate immune and inflammatory cytokines [26]. Therefore, our data extend upon previous work to show that the TLR4 signaling pathway is the upstream initiator for NLRP3 inflammasome activation, resulting in the release of pro-inflammatory cytokines in ICH.

Moreover, these pro-inflammatory cytokines induced neuronal apoptosis by enhancing the ratio of Bax and Bcl-2 in N2a cells, providing an explanation for the cognitive decline seen in ICH patients. There is evidence that suggests that *TLR4* knockout mice have significantly decreased neurological defect scores, BWC, and expression levels of inflammatory factors such as IL-10, TNF-α, and IL-1β and apoptotic protein Bax, whereas anti-apoptotic protein Bcl-2 expression is significantly increased in contrast to that in ICH mice [32]. Additionally, ICH animals lacking *TLR4* showed significantly alleviated encephaledema and nerve function impairment and reduced inflammatory factor release and cell apoptosis after ICH [32]. These studies support our results that atorvastatin ameliorated the BWC-related and cognitive defects by inhibiting neuroinflammation-induced neuronal loss after ICH. Similar to that in *TLR4* knockout models, infiltration of monocytes and neutrophil granulocytes in mice reduced peripheral inflammation and improved recovery [46]. Interestingly, the activation of the TLR4 signaling pathway may negatively regulate CD36 expression, which consequently weakens the body’s ability to clear the hematoma [23, 47]. However, the precise underlying mechanism remains unclear. TLR4 could mediate inflammatory damage after ICH, such that targeted suppression of TLR4 expression might help delay and control the development of ICH [32].

To further explore whether atorvastatin plays a protective role through TLR4, ICH mice were treated with atorvastatin showing that the BWC, neurological defects scores, and levels of inflammatory factors and apoptotic proteins in atorvastatin-treated, ICH-induced mice exhibited significant improvement, suggesting its efficacy in treating ICH and protective effect on the brain. Noteworthy, atorvastatin markedly decreased the BWC in IHC mice, suggesting that atorvastatin treatment alleviated brain edema. In agreement with these animal studies, atorvastatin is potentially efficacy in treating ICH patients by depressing NLRP3 inflammasome [48]. Therefore, we speculate that the effects of atorvastatin include protecting neurological function and reducing the inflammatory response and neuronal apoptosis via the TLR4, MyD88, and NLRP3 signaling pathways.

## References

[1] van Asch CJ, Luitse MJ, Rinkel GJ, van der Tweel I, Algra A, Klijn CJ. Incidence, case fatality, and functional outcome of intracerebral haemorrhage over time, according to age, sex, and ethnic origin: a systematic review and meta-analysis. Lancet Neurol. 2010; 9:167–76. 10.1016/S1474-4422(09)70340-0 20056489

[2] Hong KS, Kim BJ, Lee JY, Kwon SU, and PICASSO Investigators. Rationale and design of the PreventIon of CArdiovascular events in iSchemic Stroke patients with high risk of cerebral hemOrrhage (PICASSO) study: A randomized controlled trial. Int J Stroke. 2015; 10:1153–8. 10.1111/ijs.12519 26044566

[3] Pan W, Yan Q, Qin M, Jin G, Sun J, Ning X, Zhuang W, Peng B, Li G. Detection of cerebral hemorrhage in rabbits by time-difference magnetic inductive phase shift spectroscopy. PLoS One. 2015; 10:e0128127. 10.1371/journal.pone.0128127 26001112 PMC4441421

[4] Babu R, Bagley JH, Di C, Friedman AH, Adamson C. Thrombin and hemin as central factors in the mechanisms of intracerebral hemorrhage-induced secondary brain injury and as potential targets for intervention. Neurosurg Focus. 2012; 32:E8. 10.3171/2012.1.FOCUS11366 22463118

[5] Zhou Y, Wang Y, Wang J, Anne Stetler R, Yang QW. Inflammation in intracerebral hemorrhage: from mechanisms to clinical translation. Prog Neurobiol. 2014; 115:25–44. 10.1016/j.pneurobio.2013.11.003 24291544

[6] Ye L, Gao L, Cheng H. Inflammatory Profiles of the Interleukin Family and Network in Cerebral Hemorrhage. Cell Mol Neurobiol. 2018; 38:1321–33. 10.1007/s10571-018-0601-x 30027390 PMC11481843

[7] Wang J, Doré S. Inflammation after intracerebral hemorrhage. J Cereb Blood Flow Metab. 2007; 27:894–908. 10.1038/sj.jcbfm.9600403 17033693

[8] Wang X, Arima H, Heeley E, Delcourt C, Huang Y, Wang J, Stapf C, Robinson T, Woodward M, Chalmers J, Anderson CS, and INTERACT2 Investigators. Magnitude of blood pressure reduction and clinical outcomes in acute intracerebral hemorrhage: intensive blood pressure reduction in acute cerebral hemorrhage trial study. Hypertension. 2015; 65:1026–32. 10.1161/HYPERTENSIONAHA.114.05044 25801872

[9] Stutz A, Golenbock DT, Latz E. Inflammasomes: too big to miss. J Clin Invest. 2009; 119:3502–11. 10.1172/JCI40599 19955661 PMC2786809

[10] Menu P, Vince JE. The NLRP3 inflammasome in health and disease: the good, the bad and the ugly. Clin Exp Immunol. 2011; 166:1–15. 10.1111/j.1365-2249.2011.04440.x 21762124 PMC3193914

[11] Davis BK, Wen H, Ting JP. The inflammasome NLRs in immunity, inflammation, and associated diseases. Annu Rev Immunol. 2011; 29:707–35. 10.1146/annurev-immunol-031210-101405 21219188 PMC4067317

[12] Lamkanfi M, Kanneganti TD. Nlrp3: an immune sensor of cellular stress and infection. Int J Biochem Cell Biol. 2010; 42:792–5. 10.1016/j.biocel.2010.01.008 20079456 PMC2862759

[13] Antonopoulos C, Russo HM, El Sanadi C, Martin BN, Li X, Kaiser WJ, Mocarski ES, Dubyak GR. Caspase-8 as an Effector and Regulator of NLRP3 Inflammasome Signaling. J Biol Chem. 2015; 290:20167–84. 10.1074/jbc.M115.652321 26100631 PMC4536427

[14] Ma Q, Chen S, Hu Q, Feng H, Zhang JH, Tang J. NLRP3 inflammasome contributes to inflammation after intracerebral hemorrhage. Ann Neurol. 2014; 75:209–19. 10.1002/ana.24070 24273204 PMC4386653

[15] Ren H, Kong Y, Liu Z, Zang D, Yang X, Wood K, Li M, Liu Q. Selective NLRP3 (Pyrin Domain-Containing Protein 3) Inflammasome Inhibitor Reduces Brain Injury After Intracerebral Hemorrhage. Stroke. 2018; 49:184–92. 10.1161/STROKEAHA.117.018904 29212744 PMC5753818

[16] Yuan B, Shen H, Lin L, Su T, Zhong S, Yang Z. Recombinant adenovirus encoding NLRP3 RNAi attenuate inflammation and brain injury after intracerebral hemorrhage. J Neuroimmunol. 2015; 287:71–5. 10.1016/j.jneuroim.2015.08.002 26439964

[17] Kong F, Ye B, Cao J, Cai X, Lin L, Huang S, Huang W, Huang Z. Curcumin Represses NLRP3 Inflammasome Activation via TLR4/MyD88/NF-κB and P2X7R Signaling in PMA-Induced Macrophages. Front Pharmacol. 2016; 7:369. 10.3389/fphar.2016.00369 27777559 PMC5056188

[18] Kong F, Ye B, Lin L, Cai X, Huang W, Huang Z. Atorvastatin suppresses NLRP3 inflammasome activation via TLR4/MyD88/NF-κB signaling in PMA-stimulated THP-1 monocytes. Biomed Pharmacother. 2016; 82:167–72. 10.1016/j.biopha.2016.04.043 27470352

[19] Ciaramelli C, Calabrese V, Sestito SE, Pérez-Regidor L, Klett J, Oblak A, Jerala R, Piazza M, Martín-Santamaría S, Peri F. Glycolipid-based TLR4 Modulators and Fluorescent Probes: Rational Design, Synthesis, and Biological Properties. Chem Biol Drug Des. 2016; 88:217–29. 10.1111/cbdd.12749 26896420

[20] Hu QP, Mao DA. Histone deacetylase inhibitor SAHA attenuates post-seizure hippocampal microglia TLR4/MYD88 signaling and inhibits TLR4 gene expression via histone acetylation. BMC Neurosci. 2016; 17:22. 10.1186/s12868-016-0264-9 27193049 PMC4872358

[21] Gao Y, Zhuang Z, Lu Y, Tao T, Zhou Y, Liu G, Wang H, Zhang D, Wu L, Dai H, Li W, Hang C. Curcumin Mitigates Neuro-Inflammation by Modulating Microglia Polarization Through Inhibiting TLR4 Axis Signaling Pathway Following Experimental Subarachnoid Hemorrhage. Front Neurosci. 2019; 13:1223. 10.3389/fnins.2019.01223 31803007 PMC6872970

[22] Ye Y, Jin T, Zhang X, Zeng Z, Ye B, Wang J, Zhong Y, Xiong X, Gu L. Meisoindigo Protects Against Focal Cerebral Ischemia-Reperfusion Injury by Inhibiting NLRP3 Inflammasome Activation and Regulating Microglia/Macrophage Polarization via TLR4/NF-κB Signaling Pathway. Front Cell Neurosci. 2019; 13:553. 10.3389/fncel.2019.00553 31920554 PMC6930809

[23] Fang H, Chen J, Lin S, Wang P, Wang Y, Xiong X, Yang Q. CD36-mediated hematoma absorption following intracerebral hemorrhage: negative regulation by TLR4 signaling. J Immunol. 2014; 192:5984–92. 10.4049/jimmunol.1400054 24808360 PMC4049082

[24] Lin S, Yin Q, Zhong Q, Lv FL, Zhou Y, Li JQ, Wang JZ, Su BY, Yang QW. Heme activates TLR4-mediated inflammatory injury via MyD88/TRIF signaling pathway in intracerebral hemorrhage. J Neuroinflammation. 2012; 9:46. 10.1186/1742-2094-9-46 22394415 PMC3344687

[25] Liu DL, Zhao LX, Zhang S, Du JR. Peroxiredoxin 1-mediated activation of TLR4/NF-κB pathway contributes to neuroinflammatory injury in intracerebral hemorrhage. Int Immunopharmacol. 2016; 41:82–9. 10.1016/j.intimp.2016.10.025 27821296

[26] Fei X, He Y, Chen J, Man W, Chen C, Sun K, Ding B, Wang C, Xu R. The role of Toll-like receptor 4 in apoptosis of brain tissue after induction of intracerebral hemorrhage. J Neuroinflammation. 2019; 16:234. 10.1186/s12974-019-1634-x 31771613 PMC6880548

[27] Tschoe C, Bushnell CD, Duncan PW, Alexander-Miller MA, Wolfe SQ. Neuroinflammation after Intracerebral Hemorrhage and Potential Therapeutic Targets. J Stroke. 2020; 22:29–46. 10.5853/jos.2019.02236 32027790 PMC7005353

[28] Ii M, Losordo DW. Statins and the endothelium. Vascul Pharmacol. 2007; 46:1–9. 10.1016/j.vph.2006.06.012 16920035

[29] Liao JK, Laufs U. Pleiotropic effects of statins. Annu Rev Pharmacol Toxicol. 2005; 45:89–118. 10.1146/annurev.pharmtox.45.120403.095748 15822172 PMC2694580

[30] Crisby M, Nordin-Fredriksson G, Shah PK, Yano J, Zhu J, Nilsson J. Pravastatin treatment increases collagen content and decreases lipid content, inflammation, metalloproteinases, and cell death in human carotid plaques: implications for plaque stabilization. Circulation. 2001; 103:926–33. 10.1161/01.cir.103.7.926 11181465

[31] Zhao G, Yu YM, Kaneki M, Bonab AA, Tompkins RG, Fischman AJ. Simvastatin reduces burn injury-induced splenic apoptosis via downregulation of the TNF-α/NF-κB pathway. Ann Surg. 2015; 261:1006–12. 10.1097/SLA.0000000000000764 24950285 PMC4272342

[32] Chen W, Hu YQ, Jiang LF, Wu L. Mechanism of action of Zhuyu Annao pill in mice with cerebral intrahemorrhage based on TLR4. Asian Pac J Trop Med. 2016; 9:1095–100. 10.1016/j.apjtm.2016.09.004 27890371

[33] Sun Z, Wu K, Gu L, Huang L, Zhuge Q, Yang S, Wang Z. IGF-1R stimulation alters microglial polarization via TLR4/NF-κB pathway after cerebral hemorrhage in mice. Brain Res Bull. 2020; 164:221–34. 10.1016/j.brainresbull.2020.08.026 32871240

[34] Zhao J, Zhang H, Huang Y, Wang H, Wang S, Zhao C, Liang Y, Yang N. Bay11-7082 attenuates murine lupus nephritis via inhibiting NLRP3 inflammasome and NF-κB activation. Int Immunopharmacol. 2013; 17:116–22. 10.1016/j.intimp.2013.05.027 23770281

[35] Kumar A, Negi G, Sharma SS. JSH-23 targets nuclear factor-kappa B and reverses various deficits in experimental diabetic neuropathy: effect on neuroinflammation and antioxidant defence. Diabetes Obes Metab. 2011; 13:750–8. 10.1111/j.1463-1326.2011.01402.x 21447040

[36] Lamkanfi M, Dixit VM. The inflammasomes. PLoS Pathog. 2009; 5:e1000510. 10.1371/journal.ppat.1000510 20041168 PMC2791419

[37] Luo B, Li B, Wang W, Liu X, Liu X, Xia Y, Zhang C, Zhang Y, Zhang M, An F. Rosuvastatin alleviates diabetic cardiomyopathy by inhibiting NLRP3 inflammasome and MAPK pathways in a type 2 diabetes rat model. Cardiovasc Drugs Ther. 2014; 28:33–43. 10.1007/s10557-013-6498-1 24254031

[38] Xu JF, Washko GR, Nakahira K, Hatabu H, Patel AS, Fernandez IE, Nishino M, Okajima Y, Yamashiro T, Ross JC, Estépar RS, Diaz AA, Li HP, et al, and COPDGene Investigators. Statins and pulmonary fibrosis: the potential role of NLRP3 inflammasome activation. Am J Respir Crit Care Med. 2012; 185:547–56. 10.1164/rccm.201108-1574OC 22246178 PMC3297101

[39] Liao YH, Lin YC, Tsao ST, Lin YC, Yang AJ, Huang CT, Huang KC, Lin WW. HMG-CoA reductase inhibitors activate caspase-1 in human monocytes depending on ATP release and P2X7 activation. J Leukoc Biol. 2013; 93:289–99. 10.1189/jlb.0812409 23159926

[40] Massonnet B, Normand S, Moschitz R, Delwail A, Favot L, Garcia M, Bourmeyster N, Cuisset L, Grateau G, Morel F, Silvain C, Lecron JC. Pharmacological inhibitors of the mevalonate pathway activate pro-IL-1 processing and IL-1 release by human monocytes. Eur Cytokine Netw. 2009; 20:112–20. 10.1684/ecn.2009.0162 19825520

[41] Bauernfeind FG, Horvath G, Stutz A, Alnemri ES, MacDonald K, Speert D, Fernandes-Alnemri T, Wu J, Monks BG, Fitzgerald KA, Hornung V, Latz E. Cutting edge: NF-kappaB activating pattern recognition and cytokine receptors license NLRP3 inflammasome activation by regulating NLRP3 expression. J Immunol. 2009; 183:787–91. 10.4049/jimmunol.0901363 19570822 PMC2824855

[42] Barton GM, Medzhitov R. Toll-like receptor signaling pathways. Science. 2003; 300:1524–5. 10.1126/science.1085536 12791976

[43] Tak PP, Firestein GS. NF-kappaB: a key role in inflammatory diseases. J Clin Invest. 2001; 107:7–11. 10.1172/JCI11830 11134171 PMC198552

[44] Hiscott J, Marois J, Garoufalis J, D'Addario M, Roulston A, Kwan I, Pepin N, Lacoste J, Nguyen H, Bensi G. Characterization of a functional NF-kappa B site in the human interleukin 1 beta promoter: evidence for a positive autoregulatory loop. Mol Cell Biol. 1993; 13:6231–40. 10.1128/mcb.13.10.6231-6240.1993 8413223 PMC364682

[45] Son YH, Jeong YT, Lee KA, Choi KH, Kim SM, Rhim BY, Kim K. Roles of MAPK and NF-kappaB in interleukin-6 induction by lipopolysaccharide in vascular smooth muscle cells. J Cardiovasc Pharmacol. 2008; 51:71–7. 10.1097/FJC.0b013e31815bd23d 18209571

[46] Wang YC, Wang PF, Fang H, Chen J, Xiong XY, Yang QW. Toll-like receptor 4 antagonist attenuates intracerebral hemorrhage-induced brain injury. Stroke. 2013; 44:2545–52. 10.1161/STROKEAHA.113.001038 23839500

[47] Chávez-Sánchez L, Garza-Reyes MG, Espinosa-Luna JE, Chávez-Rueda K, Legorreta-Haquet MV, Blanco-Favela F. The role of TLR2, TLR4 and CD36 in macrophage activation and foam cell formation in response to oxLDL in humans. Hum Immunol. 2014; 75:322–9. 10.1016/j.humimm.2014.01.012 24486576

[48] Satoh M, Tabuchi T, Itoh T, Nakamura M. NLRP3 inflammasome activation in coronary artery disease: results from prospective and randomized study of treatment with atorvastatin or rosuvastatin. Clin Sci (Lond). 2014; 126:233–41. 10.1042/CS20130043 23944632

